# Carotenoids: How Effective Are They to Prevent Age-Related Diseases?

**DOI:** 10.3390/molecules24091801

**Published:** 2019-05-09

**Authors:** Bee Ling Tan, Mohd Esa Norhaizan

**Affiliations:** 1 Department of Nutrition and Dietetics, Faculty of Medicine and Health Sciences, Universiti Putra Malaysia, Serdang 43400, Selangor, Malaysia; tbeeling87@gmail.com; 2 Laboratory of Molecular Biomedicine, Institute of Bioscience, Universiti Putra Malaysia, Serdang 43400, Selangor, Malaysia; 3 Research Centre of Excellent, Nutrition and Non-Communicable Diseases (NNCD), Faculty of Medicine and Health Sciences, Universiti Putra Malaysia, Serdang 43400, Selangor, Malaysia

**Keywords:** aging, cancer, cardiovascular disease, dementia, diabetes, inflammation, oxidative stress

## Abstract

Despite an increase in life expectancy that indicates positive human development, a new challenge is arising. Aging is positively associated with biological and cognitive degeneration, for instance cognitive decline, psychological impairment, and physical frailty. The elderly population is prone to oxidative stress due to the inefficiency of their endogenous antioxidant systems. As many studies showed an inverse relationship between carotenoids and age-related diseases (ARD) by reducing oxidative stress through interrupting the propagation of free radicals, carotenoid has been foreseen as a potential intervention for age-associated pathologies. Therefore, the role of carotenoids that counteract oxidative stress and promote healthy aging is worthy of further discussion. In this review, we discussed the underlying mechanisms of carotenoids involved in the prevention of ARD. Collectively, understanding the role of carotenoids in ARD would provide insights into a potential intervention that may affect the aging process, and subsequently promote healthy longevity.

## 1. Introduction

The average life expectancy has been rising rapidly in recent decades, with an average of 72.0 years in 2016 globally [1]. However, the healthy life expectancy was 63.3 years in 2016 worldwide [1]. In view of the demographics of the global population from 2000 to 2050, the population aged 60 years or more is estimated to increase from 605 million to 2 billion people [2]. In many countries, the average life expectancy aged 60 years could expect to live another 20.5 years in 2016 [1]. This longevity accounts for a growing share of age-related diseases (ARD) and their consequent economic and social burden [3]. In fact, aging is positively associated with biological and cognitive degeneration including cognitive decline, psychological impairment, and physical frailty [4].

Reactive oxygen species (ROS) are continuously generated in normal aerobic metabolism as a by-product; however, when the amount is elevated under stress, it may cause potential biological damage [5]. Oxidative stress emerges from an imbalance of either pro- and/or antioxidant molecules, being characterized by the decreased capacity of endogenous systems to combat an oxidative attack and subsequently leading to molecular and cellular damage [6]. Oxidative stress has been recognized as the main contributor to the pathophysiology and pathogenesis of ARD [7] such as metabolic syndromes, atherosclerosis, osteoporosis, obesity, dementia, diabetes, cancer, and arthritis [8,9].

ARD have become the most common health threats in recent decades. ARD have been linked to structural changes in mitochondria, accompanied by an alteration of biophysical properties of the membrane such as reduced fluidity and altered electron transport chain complex activity, which in turn contribute to mitochondrial failure and energy imbalance. This perturbation impairs mitochondrial function and cellular homeostasis, and increases susceptibility to oxidative stress [10,11]. The elderly population is susceptible to oxidative stress due to the inefficiency of their endogenous antioxidant systems [12]. An irreversible progression of oxidative decay due to ROS also causes a negative impact on the biology of aging such as reducing lifespan, increasing disease incidence, and the impairment of physiological functions [13]. Several organs, for example the heart and brain, with a high consumption of oxygen and limited replication rate are vulnerable to these phenomena, suggesting the high prevalence of neurological disorders and cardiovascular disease (CVD) in elderly populations [14,15]. Increased ROS has been linked to the progression and onset of aging. Although ROS generation may not be an essential factor for aging [16], they are more likely to aggravate ARD development through interaction with mitochondria and cause oxidative damage [17]. Due to their reactivity, high levels of ROS can generate oxidative stress by interrupting the balance of prooxidant and antioxidant levels [18]. Substantial evidence highlights that carotenoids can decrease oxidative stress and the progression of ARD [19]. Lycopene, a carotenoid that is abundantly found in tomatoes, is a crucial antioxidant source. A meta-analysis study has demonstrated an inverse relationship between lycopene intake and cardiovascular disease (CVD) risk [20]. This favorable effect could be attributed to the decreased inflammatory response and cholesterol level, as well as the reduced oxidation of biomolecules [21]. Besides CVD, several studies have also found that consumption of carotenoid-rich fruits and vegetables can prevent cancers such as prostate and cervical [22,23,24]. As many studies show that carotenoid intake is negatively associated with ARD by disrupting the formation of free radicals and subsequently reduces oxidative stress, carotenoid has been foreseen as a promising nutritional approach for ARD. Therefore, the role of carotenoids that combat oxidative stress and promote healthy longevity is worth to discuss further. Of particular interest in this review, we discussed the underlying mechanisms of carotenoids involved in the prevention of ARD. Understanding the role of carotenoids in ARD would provide insight for potential interventions that may affect the aging process, and subsequently promoting healthy longevity.

## 2. Carotenoids

Carotenoids are a family of naturally occurring organic pigmented compounds that are produced by the fungi, several bacteria, and plastids of algae and plants [25]. Notably, red pea aphid (*Acyrthosiphon pisum*) and spider mite (*Tetranychus urticae*) are the only animals that produce carotenoids from fungi through gene transfer [26]. In plants, carotenoids contribute to the photosynthetic machinery and protect them from photo-damage [27]. They occur in all organisms capable of photosynthesis, a process to convert into chemical energy in the presence of sunlight. Generally, carotenoids absorb wavelengths between 400 and 550 nanometers, and hence the compounds are present in red, orange, or yellow color [28].

Nearly 600 carotenoids have been identified in nature to modulate a broad spectrum of functions [29]. However, only about 50 carotenoids are found in a typical human diet [30], while about 20 carotenoids are present in human tissues and blood [31]. Carotenoids are classified into two groups, namely xanthophylls and carotenes, according to their chemical constituents [32]. Oxygenated derivatives are known as xanthophylls; while hydrocarbon only carotenoids (lycopene, β-carotene, and α-carotene) are called carotenes. Additionally, aldehyde groups (β-citraurin), epoxide groups (neoxanthin, antheraxanthin, and violaxanthin), oxo/keto groups (canthaxanthin and echinenone), and oxygen substituents (zeaxanthin and lutein) are categorized as complex xanthophylls [33].

## 3. Chemical Structures

**Figure 1:**
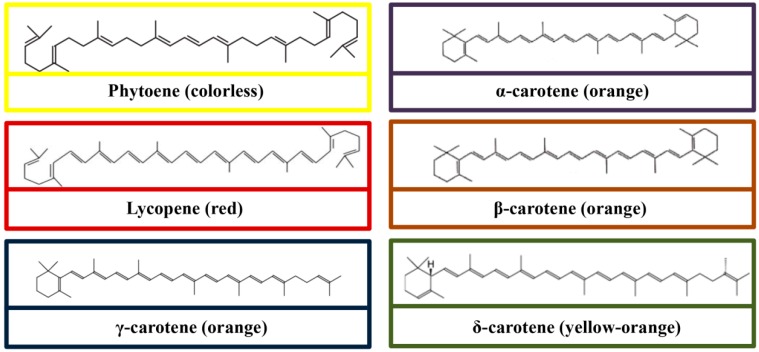
Molecular structures of carotenes (Phytoene, lycopene, γ-carotene, α-carotene, β-carotene, and δ-carotene).

**Figure 2:**
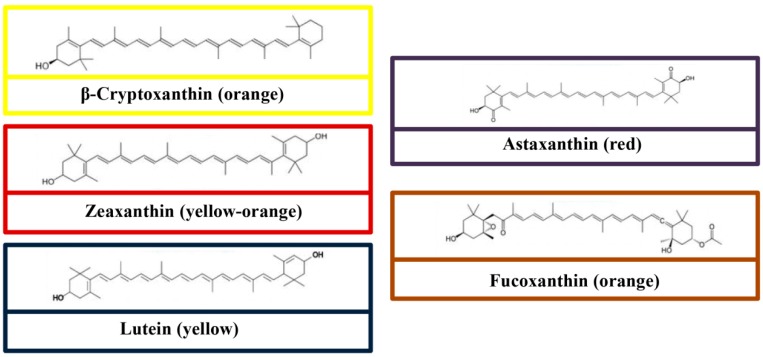
Molecular structures of some common xanthophylls (β-cryptoxanthin, zeaxanthin, lutein, astaxanthin, and fucoxanthin).

In particular, most of the carotenoids are tetraterpenoids, containing 40 carbon atoms and derived from eight isoprene molecules [34]. All carotenoids have a polyisoprenoid structure, accompanied by a long-conjugated chain adjacent with multiple double bonds and symmetry on the central double bonds. The molecular structures of carotenes and xanthophylls are shown in Figure 1 and Figure 2, respectively. Alteration of the basic acyclic structure acquired oxygen-rich functional groups [35]. One of the features of carotenoid is a strong coloration, which is a consequence of light absorption in the presence of a conjugated chain [36]. Due to the presence of the electron-rich conjugated system of the polyene structure, carotenoids scavenge the free radicals by trapping peroxyl radicals and quenching the singlet oxygen [37]. Indeed, the conjugated double bond is critical for the proper functioning of carotenoids, for example in light absorption for photosynthetic organisms [36].

## 4. Dietary Sources

Carotenoids are abundantly found in deeply pigmented fruits and vegetables (Table 1), in which the orange-yellow vegetables and fruits are rich in β-carotene and α-carotene. While, α-cryptoxanthin, lycopene, and lutein are found in orange fruits, tomatoes and tomato products, and dark green vegetables, respectively [38]. Egg yolk is a highly bioavailable source of zeaxanthin and lutein [39]. The unsaturated nature of the carotenoids makes them prone to oxidation [40]. Other factors like pH, light, and temperature can also affect the color and nutritional value of foods [41]. Some common household cooking methods, for example boiling, steaming, and microwave cooking, do not markedly change the extent of the carotenoid content in food [42]. However, extreme heat can cause oxidative damage to carotenoids [42].

## 5. Metabolism and Bioavailability

There are several factors that affect the carotenoid absorption, bioavailability, breakdown, transport, and storage. For example, the dietary intake of fat (in the form of salad dressing, cooking oil for instance extra virgin olive oil or whole egg) at the same meal with carotenoid consumption (cooked vegetables or raw vegetable salad) has been found to effectively increase the absorption of some carotenoids [56,57,58,59]. The bioavailability of carotenoids may reduce when consumed within the same meal due to the competition between carotenoids during absorption [60]. In addition, dietary fiber from plant sources, for example guar gum and pectin, were found to decrease carotenoid absorption [61], and the localization of carotenoids with the chromoplasts and chloroplasts of plants may reduce the bioavailability [62]. A study reported by Hornero-Mendez and Mínguez-Mosquera [63] evaluated the impact of cooking on carotenoids in the plant. The data showed that although heat reduces the carotenoid content, the bioavailability of the carotenoids was enhanced compared to the control (uncooked) [63]. Furthermore, Baskaran et al. [64] evaluated the micellar phospholipid in relation to the intestinal uptake of carotenoids in in vivo study. The data showed that phosphatidylcholine suppressed the accumulation of lutein and β-carotene in plasma and liver, suggesting the phospholipids derived from food and bile could influence the cellular uptake of carotenoids solubilized in mixed micelles formed in the intestinal tract. In addition, the rate of bioaccessibility of carotenoids is highly affected by the food matrix. The previous study revealed that in vitro transfer rate of β-cryptoxanthin, zeaxanthin, and lutein is nearly 100% from fruits such as sweet potato, grapefruit, kiwi, and orange compared to the vegetables such as spinach and broccoli, which is between 19 to 38% [65]. This observation indicates that the release of carotenoids from a food matrix followed by absorption is a determining factor for delivering potential health benefits.

The release of carotenoids from the food matrix is highly dependent on their state, as well as their associations with other food components such as protein [66]. As an example, the microcrystalline form of carotenoids, for instance lycopene in tomato and β-carotene in carrot, reduces their bioavailability compared to those that are immersed entirely in lipid droplets [36]. The bioavailability of carotenoids is markedly varied in food. The previous data stated that nearly 5% of carotenoids (whole, raw vegetables) are absorbed by the intestine whereas up to 50% of the carotenoid is absorbed from the micellar solutions [67]. This finding implies that the physical form of carotenoids present in intestinal mucosal cells is vitally important. Many studies have revealed that thermal treatment increases the bioaccessibility of carotenoids and improves their absorption due to the bond loosening and disruption of cell walls [68]. They are absorbed into gastrointestinal mucosal cells and remain unchanged in the tissues and circulations [69,70]. In the intestine, carotenoids are absorbed via passive diffusion after being incorporated into the micelles formed by the bile acid and dietary fat. Subsequently, these micellular carotenoids are incorporated into the chylomicrons and released into the lymphatic system. Ultimately, they bind with the lipoprotein at the liver and are released into the bloodstream [71]. Carotenoids are predominantly accumulated in adipose tissue and the liver; whereas in brain stem tissue, the carotenoid concentration is below the detection limit [72,73]. Other factors such as gender, aging, nutritional status, genetic factor, and infection may also influence the bioavailability of carotenoids [74,75]. It has been demonstrated that any disease with an abnormal absorption of fat from the digestive tract markedly alters the incorporation of carotenoids. Additionally, interaction with drugs such as aspirin and sulphonamides has been found to reduce the bioavailability of β-carotene [74].

## 6. Physiological Changes in Aging

Aging is characterized by a progressive loss and decline of tissues and organ systems. The degeneration rate is varied between individuals and is highly dependent on genetics and environmental factors, for instance exercise, ionizing radiation, pollutant exposure, and diet. In general, the physiological changes of aging are divided into three groups that include (1) changes in cellular homeostatic mechanisms, such as extracellular fluid volume, blood, and body temperature; (2) a decrease in organ mass; and (3) the loss and decline of the functional reserve of the body system [76]. The loss of functional reserve may impair the ability of an individual to cope with external challenges, for instance trauma and surgery.

Cardiovascular aging attenuates contractile and mechanical efficiency. The specific changes include an increase in smooth muscle tone, promotion of collagenolytic and elastolytic activity, and arterial wall thickening [77]. Subsequently, vessels stiffen progressively with age and contribute to the elevation of systolic arterial pressure and increase cardiac afterload and systemic vascular resistance. This phenomenon is usually demonstrated in isolated systolic hypertension, in which the left ventricle has to work harder to eject blood into the stiffer aorta, and hence increase the workload and contribute to the left ventricular hypertrophy. Hypertrophy of myocytes in response to increased afterload may promote contraction time as well as the cardiac cycle. Ventricular relaxation is delayed at the time of mitral valve opening and leads to diastolic dysfunction. Further, the early diastolic filling rate is also decreased with age and partly compensated by an elevated rate of late diastolic filling. Aging is also linked to the reduction of cardiac output in the face of falls in blood pressure [77].

In the context of the central nervous system, aging reduces the neural density, accounting for nearly a 30% loss of brain mass by the age of 80 years, largely grey matter. Growing older is linked to a reduction of central neurotransmitters such as acetylcholine, serotonin, and catecholamine. In addition, aging may also reduce dopamine uptake transporters and decrease γ-aminobutyric acid, β-adrenergic, α_2_-adrenergic, and cortical serotonergic binding sites. All these changes may reduce the speed of memory and processing [77].

The greatest change in gastrointestinal physiology affecting nutrient bioavailability is atrophic gastritis, which presents in nearly 20% of the elderly population [78]. It has been shown that a slight decline in the secretion of pepsin and hydrochloric acid occurs with advancing age. Nutrient absorption is affected by low acid conditions in the stomach. Research evidence revealed that growing older is associated with the age-associated decline in the absorption of certain substances absorbed by active mechanisms such as vitamin B_12_, β-carotene, iron, and calcium [79]. For example, dietary vitamin B_12_ is linked to the food protein, in which the vitamin B_12_ molecules must be digested before bound to the endogenous R binders. This digestion takes place in the presence of pepsin and acid. If stomach acid is low, the digestion of vitamin B_12_ cannot take place effectively [78].

In addition to the effects mentioned above, aging may reduce the number of fibroblasts and keratinocytes, decrease epidermal cell turnover, and impair the barrier function [80]. Moreover, aging can also decrease the vascular network such as round hair glands and bulbs (skin atrophy and fibrosis). Notably, elderly people are susceptible to the changes in cutaneous function due to the reduction in vitamin D synthesis. These changes increase their susceptibility to skin injuries such as skin tear and pressure ulcer [77].

## 7. The Role of Carotenoids in the Prevention of ARD

Antioxidant plays a predominant role in the termination of oxidative chain reactions by disrupting the free radical intermediates [81]. Antioxidants control autoxidation by disrupting the formation of free radicals or suppressing the propagation of free radicals through several mechanisms. This compound facilitates in quenching •O_2_^−^, breaking the autoxidative chain reaction, inhibiting the formation of peroxides, and scavenging the species that promote the peroxidation [82].

**Figure 3:**
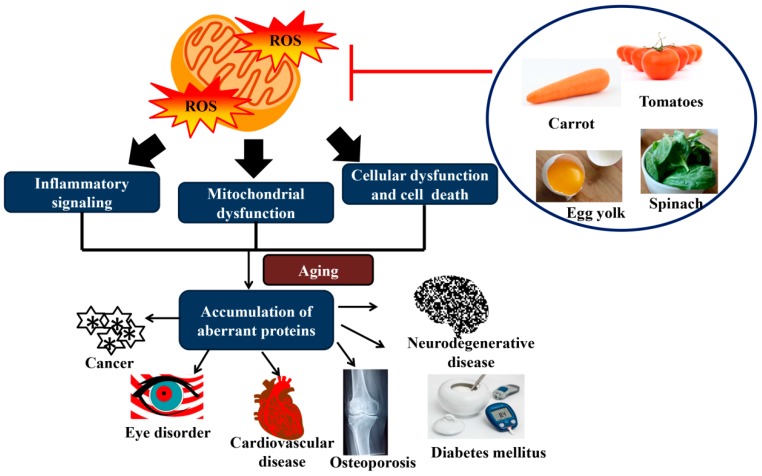
The effect of oxidative stress and the interaction of carotenoids in relation to ARD. Accumulation of reactive oxygen species (ROS) leads to inflammation, cellular dysfunction and cell death, and mitochondrial dysfunction. Mitochondria function decline, oxidative stress response in aging, and accumulation of aberrant proteins may contribute to ARD. The consumption of carotenoids may block ROS production.

Carotenoids are known as a highly effective physical and chemical singlet oxygen quencher and a potent scavenger of ROS [83]. The previous study stated that the antioxidant activity of lycopene is superior to α-tocopherol and β-carotene [84]. This favorable effect is attributed to the singlet oxygen quenching ability [85], suggesting that a tetraterpene hydrocarbon polyene accompanied with two unconjugated and eleven conjugated double bonds readily interact with electrophilic reagents, and subsequently affect the reactivity of oxygen and oxygenated free radical species [85]. The previous finding has revealed that a high consumption of carotenoids is inversely associated with ARD [86]. It has been suggested that the alleviation of chronic diseases is mainly due to the antioxidant properties of carotenoids [87]. Figure 3 shows the effect of oxidative stress and the interaction of carotenoids in relation to ARD.

### 7.1. Eye Disorders

Visual impairment has become the second most common cause of lived with disability [88]. Diabetic retinopathy, glaucoma, cataract, and age-related macular degeneration (AMD) are the most common types of vision loss among the elderly [89]. The development of AMD is not only due to the age factor, other factors, for example diet, oxidative stress, and smoking, may also increase the risk [90]. Tosini et al. [91] revealed that prolonged exposure to blue light emitted by energy-efficient lightbulbs and electronics enhanced retinal cell damage. This study further demonstrated that long-term exposure to energy-efficient lightbulbs and electronics can reduce visual function and promote AMD [91].

AMD is the predominant contributor of blindness among the elderly aged 75 years and above in developed countries [92,93]. AMD contributes approximately 8.7% of all blindness globally [94]. Notably, some research has emerged to predict that the percentage of AMD patients will double between 2010 and 2050 [95]. Non-proliferative postmitotic cells including retinal pigment epithelium cell and photoreceptors are particularly sensitive to oxidative damage due to the absence of DNA damage detection systems compared to other cells [96]. In the context of cataracts, zeaxanthin and lutein therapy has provided significant beneficial outcomes [97]. Zeaxanthin/lutein (2 mg/10 mg) significantly reduced the risk of cataract surgery [98]. Moreover, AMD is inversely correlated with the dietary intake of a carotenoid-rich diet (5–10 mg/day) compared to those individuals who rarely or never consume carotenoids [98].

Carotenoids have been demonstrated as an eye-sight protecting agent [99]. Such carotenoids are categorized as pro-vitamin A comprised of the unsubstituted β-ionone ring (γ-carotene, α-carotene, β-carotene, and β-cryptoxanthin) which can be converted into retinal [100]. Two dietary carotenoids, namely zeaxanthin and lutein, are macular pigments found in the human retina [101]. Macular pigments exert antioxidant properties, which can absorb short wavelengths and high energy blue light, and subsequently protect the retina from photochemical damage [86]. This pigment can protect against UV-induced peroxidation and neutralize ROS [101].

Deficiency of vitamin A affects immunity, which can damage the light-sensitive receptors [102]. Further, vitamin A deficiency may also lead to permanent blindness called xerophthalmia [103]. The previous study stated that supplementation with carotenoids such as zeaxanthin (2 mg/day/year) and lutein (10–20 mg/day/year) can increase macular pigment optical density levels [104,105]. Several studies reported by Hammond et al. [104] and Nolan et al. [106] also showed that zeaxanthin/lutein (2 mg/10 mg/day/year) can enhance visual performance such as photostress recovery, glare tolerance, and contrast sensitivity. Collectively, carotenoid intake could be a potential approach for the amelioration of oxidative stress and provide potential benefits for ocular health and function. The potential implication of carotenoids on AMD, as well as the dosage of the zeaxanthin and lutein when combined with other nutrients is worthy of further investigation in randomized clinical trials.

### 7.2. Neurodegenerative Diseases

Dementia is a chronic and progressive neurodegenerative disease in which there is deterioration in behavior, thinking, memory, and the ability to perform daily activities [107]. Dementia has become one of the major causes of disability and dependency among older people and contributes to nearly 60% of the total cases. It is projected that by 2050 there will be 152 million dementia cases in low- and middle-income countries [107]. Alzheimer’s disease is the most common form of dementia and accounts for nearly 60–70% of cases [107].

The data from the previous study revealed that the concentration of carotenoids is passively associated with cognitive performance in both cognitively intact and cognitively impaired people [108,109]. A human study involving 91 healthy individuals suggested that twelve months supplementation with lutein (10 mg/day), zeaxanthin (2 mg/day), and *meso*-zeaxanthin (10 mg/day) improved the memory compared to the placebo control group [110]. A study reported by Rubin et al. [111] also demonstrated that carotenoids (16 mg/day for 26 days) are inversely associated with inflammatory markers, for instance interleukin (IL)-1β, tumor necrosis factor-α (TNF-α), IL-6, vascular cell adhesion molecule-1 (VCAM-1), and monocyte chemoattractant protein 1 (MCP-1) in both human and animal models. A study analyzed of 3031 participants aged 40–75 years revealed that total carotenoids (1.63 µmol/L) were negatively correlated with retinol binding protein 4 (RBP4) [112]. RBP4 also known as adipose-derived cytokine is a sole retinol transporter in the blood which is secreted from the adipocyte and liver [113]. RBP4 plays a crucial role as a proinflammatory marker by activating c-Jun N-terminal kinase (JNK) and nuclear factor-kappa B (NF-κB) pathways [114,115], as well as increasing the secretion of IL-1β, IL-6, and TNF-α expression. Thus, controlling systemic inflammation could be a targetable tool for the prevention of ARD.

Much information indicates that carotenoids may limit neuronal damage from free radicals, which is potentially served as a modifiable risk factor for cognitive decline. The data from 2011–2014 National Health and Nutrition Examination Survey involving 2796 participants aged ≥60 years demonstrated that lutein and zeaxanthin supplementation (2.02 mg/day) may prevent cognitive decline [116]. Carotenoids delay neurodegenerative diseases progression through several pathways, for example suppress proinflammatory cytokines [117], trigger Aβ peptide production [118], and reduce oxidative stress [119]. Due to its high binding energy with Alzheimer’s disease-associated receptors (histone deacetylase and P53 kinase receptors) [120], β-carotene is potential to be an Alzheimer’ disease antagonist. Fucoxanthin, a marine carotenoid, destabilizes Aβ fibril and inhibits Aβ formation [121]. Likewise, Ono and Yamada [122] reported that both β-carotene and vitamin A can block the oligomerization of Aβ42 and Aβ40 during Aβ peptide formation. Further, lycopene (1–4 mg/kg body weight/14 days) also decreases the Aβ42-induced inflammatory cytokine, for instance TNF-α, NF-κB, IL-1β, and transforming growth factor beta (TGF-β) in the brain [123]. High serum carotenoid levels such as lycopene, zeaxanthin, and lutein were found to reduce Alzheimer’s disease mortality [124]. Collectively, carotenoids play a significant role as an antioxidant to delay the progression of neurodegenerative disease.

### 7.3. Cardiovascular Disease

According to the World Health Organization [125], nearly 17.9 million people die from CVD, represents 31% of all deaths worldwide. About 85% of all CVD deaths are due to strokes and heart attacks [125]. CVD is the disorder of blood vessels and the heart such as cerebrovascular disease, rheumatic heart disease, and coronary heart disease [125]. CVD is the major clinical concern in the elderly, with 68% of individuals aged 60–79 years having CVD and the prevalence is increased to 85% among people aged 80 years and above [126]. Oxidative stress is implicated in the development and progression of CVD [127]. High oxidative stress in the heart is one of the common characteristics of CVD [128]. Indeed, reduced antioxidant defense and enhanced ROS accumulation can cause systemic oxidative damage in CVD patients [129].

Carotenoids have been reported to prevent oxidative stress-induced diseases including CVD [130]. The implication of carotenoids against pathophysiology of CVD has been widely studied in both in vivo and in vitro models [131,132]. Lutein suppresses the NF-κB activation which plays a prominent role in the pathogenesis of several human diseases [133]. The anti-inflammatory and antioxidant properties of lutein (1–25 µM/24 h) reduced the risk of coronary artery disease [134] and CVD [135] in the elderly population. Lutein consumption (one soft boiled egg per day for 4 weeks) was shown to reduce the oxidized low-density lipoprotein (LDL), implies that lutein may prevent the development of atherosclerosis [136]. High plasma lutein levels were found to protect the myocardium from ischemia injury by decreasing oxidative stress and apoptosis [135]. A meta-analysis involving 387,569 participants suggested that a high lutein intake or high lutein concentration in the blood reduced the risk of stroke and coronary heart disease [137]. The previous study reported by Costa-Rodrigues et al. [138] further revealed that carotenoids (lycopene) are of benefit in the protection of vascular, endothelial, and cardiac. Moreover, research evidence also indicates that carotenoids reduce LDL-cholesterol plasma levels [139] and promote high-density lipoprotein (HDL) functionality (three eggs for 30 days) [140]. Compared to those who rarely or deficient in lycopene, individuals who supplemented with lycopene may trigger a significant reduction in coronary artery disease [141]. Although most of the studies have reported a positive effect of lycopene on cardiovascular health, not all data demonstrated such a link. Several human intervention studies failed to identify an inverse relationship between lycopene intake and CVD markers [140,142,143,144,145]. There are many reasons underlying these negative associations. Both the metabolism and bioavailability of lycopene are highly affected by genetic variability, as they are found in more than 28 single nucleotide polymorphisms in 16 genes [146,147]. In addition, the cardiovascular markers utilized in different studies also varied significantly, which makes detailed comparisons difficult. A difference in lycopene sources and doses may also reduce the lycopene effects, which in turn influence the observed effects. Further, most of the studies used less than 100 subjects, which reduce the statistical power of the results. Therefore, further studies should be performed in large populations, preferably from the same geographic location to avoid high genetic variability. The processing method and amount of tomatoes ingested also should be strictly controlled [138]. Taken together, carotenoid intake might be a promising strategy to enhance cardiovascular health.

### 7.4. Cancer

Cancer represents the second most common cause of death worldwide, with nearly 9.6 million deaths and 18.1 million new cases in 2018 [148,149]. Emerging research evidence has suggested that 30–50% of cancer deaths could be prevented by modifying the key risk factors, for instance exercise regularly, maintaining healthy body weight, reducing alcohol consumption, and avoiding tobacco [148].

Carotenoids have been reported to decrease the risk of certain cancers such as colon [150], prostate [151], and lung [152]. Several carotenoids, for instance lutein, zeaxanthin, and lycopene, have been reported to decrease the inflammatory mediator’s production through the blockage of NF-κB pathway [153,154]. Lutein was found to negatively link to several types of cancer. A study obtained by Chang et al. [133] reported that lutein decreases the proliferation of breast cancer cells, ameliorates ROS, and improves the expression of cellular antioxidant enzymes via activation of nuclear factor E2-related factor 2 (Nrf2)/antioxidant responsive element (ARE) and inhibition of NF-κB pathways. In prostate cancer patients aged 64–75 years, high carrot, tomatoes, and lycopene intakes were shown to decrease the risk of prostate cancer compared to those with low carrot, tomatoes, and lycopene consumption [22]. The data from a human population-based study involving 638 independently living elderly aged 65–85 years revealed that increased serum carotenoid levels are inversely associated with cancer mortality [155]. The preventive role of carotenoids against cancer could be attributed to their antioxidant activity. In fact, the anticancer ability of carotenoids such as lycopene is modulated via several mechanisms such as apoptosis, cell cycle arrest, phase II detoxifying enzymes, and growth factor signaling [156]. However, a previous study revealed that smokers who supplemented with β-carotene (20 mg/day for 5–8 years) experienced increased lung cancer incidence, and these findings were not associated with the nicotine or tar level of cigarettes smoked, suggesting that all smokers should continue to avoid β-carotene supplementation [157]. The detrimental effect of β-carotene supplementation in smokers could be due to the instability of the β-carotene molecule in the lung after exposure to cigarette smoke. Oxidized β-carotene metabolites diminish retinoic acid levels and thus enhance lung carcinogenesis [158]. Taken together, regular consumption of carotenoids may become a useful approach to ameliorate oxidative stress. The beneficial effect of carotenoids in relation to cancer is worth attention.

### 7.5. Diabetes Mellitus

Diabetes mellitus is a chronic disease due to the deficiency or ineffective of the pancreas to produce insulin. The prevalence of diabetes has risen from 108 million in 1980 to 422 million in 2014 [159]. Nearly 1.6 million people worldwide died due to diabetes in 2016 [159]. Type 2 diabetes is the most common form of the disease, accounting nearly 90% of all diabetes mellitus cases worldwide [159]. Diabetes mellitus is a progressive disease, accompanied by complications including macro- and microvascular damage, neuropathy, retinopathy, and nephropathy [160].

Oxidative stress has been recognized as a key risk factor in the development of diabetes [161]. Several risk factors for instance aging, obesity, and unhealthy dietary intake, all contribute to an oxidative environment and subsequently alter the insulin sensitivity via impairment of glucose tolerance or promote the insulin resistance [162]. Hyperglycemia is commonly related to diabetes and leads to the progression and an overall oxidative environment [163]. The dysregulation of cellular and molecular process is common in type 2 diabetes, particularly in β-cells. Reactive nitrogen species (RNS) and ROS, for instance hydroxyl radical (OH∙), peroxynitrite (ONOO−), NO, superoxide anion (O_2_^•−^), and H_2_O_2_, all contribute to key metabolic and physiologic processes [164,165].

Another common carotenoid, astaxanthin, is a potent antioxidant for the prevention and treatment of diabetes. An animal study has shown that astaxanthin (1.0 mg/mouse/day for 13 weeks) decreases blood glucose levels, improves insulin serum levels, and reduces glucose tolerance in type 2 diabetes mellitus rodent models [166]. A 10-year prospective study involving 37,846 men and women revealed that high dietary intake of β-carotene (10 *±* 4 mg/day) can reduce the risk of type 2 diabetes mellitus [167]. A low serum β-carotene level has also been associated with impaired insulin sensitivity [168]. Another common carotenoid, fucoxanthin has been demonstrated to prevent diabetes mellitus. Maeda et al. [169] revealed that feeding obese mice Fucoxanthin-rich Wakame lipids (1.06–2.22%) may restore insulin and blood glucose levels via the upregulation of glucose transporter type 4 (GLUT4) mRNA expression in the skeletal muscle. A previous study reported by Manabe et al. [170] evaluated astaxanthin in relation to inflammatory markers and proinflammatory cytokine production. The data showed that astaxanthin (10^−7^–10^−4^ M) reduces high glucose-induced ROS production in the mitochondria and downregulates the expression of cyclooxygenase-2 (COX-2), TGF-β, NF-κB, and MCP-1. In a further study focused on inflammation outcomes, Kim et al. [171] found that astaxanthin inhibits the peroxynitrite (ONOO^−^), nitric oxide (NO), and superoxide (O_2_^−^) induced by high glucose concentration. These data suggest that astaxanthin may exert the potential in the prevention of diabetic nephropathy. The Epidemiology of Vascular Aging Study involving 127 diabetes cases and 1389 volunteers aged 59–71 years revealed that individuals with high plasma carotenoid levels were significantly reduced the risk of dysglycemia [172]. Collectively, carotenoids may be a useful nutritional intervention for diabetes and its complications.

### 7.6. Osteoporosis

Osteoporosis is the most common metabolic bone disease, which is characterized by low bone mass and increase bone fragility [173]. Osteoporosis has become a global epidemic, affecting more than 8.9 million fractures annually worldwide [174]. Nearly 75% of the distal forearm, spine, and hip fractures occur in patients aged 65 years and above [175]. By 2050, the incidence of hip fracture is expected to increase by 240% and 310% in women and men, respectively [176].

Studies in both in vivo and in vitro models have suggested that carotenoids could prevent bone loss via the reduction of oxidative stress. Osteoclastogenesis and the apoptosis of osteocytes and osteoblasts are accelerated with the presence of oxidative stress, and subsequently lead to bone resorption [177,178]. A study found that a high intake of β-carotene, β-cryptoxanthin, and lutein/zeaxanthin reduces the risk of hip fracture in the middle-aged and elderly population [179]. Further, epidemiological studies have also found that a dietary intake of carotenoids may decrease the risk of osteoporosis [180] and improve bone mineral density [181]. The in vivo study further demonstrated that lutein (50 mg/kg for 4 weeks) protects the ovariectomized rats against oxidative stress and osteoporosis by downregulating the inflammation and osteoclast-specific marker (NFATc1) expression via Nrf2 activation [182]. Likewise, Tominari et al. [183] also showed that lutein (3, 10, and 30 µM) suppresses osteoclastic bone resorption and enhances bone formation. High serum lutein and zeaxanthin levels increase bone density in young healthy adults, suggesting that lutein and zeaxanthin play a pivotal role in optimal bone health [184].

## 8. Carotenoids and Aging

Numerous animal and clinical studies suggest that a diet rich in antioxidants can prevent aging [185]. In support of this, an animal study has revealed that lutein could prolong the lifespan and ameliorate the mortality rate induced by hydrogen peroxide and paraquat in *Drosophila melanogaster* [186]. The data showed that supplementation with 0.1 mg/mL lutein significantly increased the mean lifespan of Oregon-R-C (OR) wild type flies by 11.35% compared to the control group [186]. This study further revealed that the maximum lifespan is increased more than 11.23 days after supplementation with 0.1 mg/mL lutein compared to the control [186]. Similarly, the study obtained by Neena et al. [187] has also demonstrated that lutein (0.5, 1.5, 5, 15 µM) could reduce the age-associated decline in human skin cells. Despite none of the clinical study demonstrating that a diet high in lutein could promote human lifespan, several human clinical studies revealed that a dosage ranging from 2.4–30 mg/day is beneficial to human health without undesirable outcomes [188]. In another study, Yazaki et al. [189] showed that astaxanthin (0.1–1 mM) can prolong the lifespan in the wild-type and long-lived mutant *age-1* of *C. elegans*. The data revealed that astaxanthin increased DAF-16 gene expression and reduced mitochondrial production of ROS, suggesting that carotenoid is partially involved in the modulation of insulin-like growth factor 1 (IGF-1) signaling [189]. Indeed, IGF-1 plays a predominant role in biological aging [190]. Fucoxanthin (0.3–1.0 µM) has also been reported to prolong lifespan and promote the viability of the organism such as *Drosophila melanogaster* and *C. elegans* [191]. An adequate intake of lutein-rich food is vitally important throughout the lifespan. The previous finding suggests that carotenoids such as lutein play an important role in neural health (cognitive and visual function) in adults [192], implying that carotenoids may provide an optimal or better health outcome.

## 9. Safety and Toxicity

In a well-balanced diet, the intake of carotenoids, such as lutein, is sufficient to maintain health. However, supplementation is needed in cases of chronic disease or the inadequate absorption of carotenoids. Several studies conducted in both in vitro [193] and animal models [193,194] have revealed that the use of lutein is safe without teratogenic and mutagenic outcomes. Despite the fact that no toxic effect was observed during lutein supplementation in both intervention and epidemiological studies [195], the Joint Expert Committee on Food Additives established an upper safety limit for daily lutein consumption of 2 mg/kg [196]. Whereas the European Food Safety Authority (EFSA) indicated an upper safety limit of 1 mg/kg [197]. EFSA further established an upper limit for lutein-enriched milk for infants of 250 µg/L [198]. Notably, the data showed that there is no interaction between lutein consumption and cytochrome P450 enzyme activity, suggesting that lutein may not modify the metabolism of endogenous or exogenous substances [199]. An animal study has shown that mice lacking β-carotene oxygenase 2 significantly increased the mitochondrial dysfunction and oxidative stress as well as developed pathologic carotenoid accumulation [200]. This finding implied that an excessive carotenoid intake may contribute to toxicity under certain circumstances. Olmedilla et al. [201] found that the supplementation of lutein at a dosage of 15 mg/day for 20 weeks increased the risk of skin yellowing (carotenodermia). Similarly, the data from the observational study revealed that lutein may increase the risk of lung cancer, particularly non-small cell lung cancer in smokers [202]. The population-based study has also reported that lutein supplementation increased the risk of crystalline maculopathy in old women. The adverse outcomes are reversed after lutein intake discontinuation [203]. Although research has demonstrated a positive association between lutein and the risk of several diseases, the survey conducted by EFSA concluded that the data obtained were insufficient to show an adverse outcome [197]. Consistent with the data reported by EFSA, the Age-Related Eye Disease Study 2 (AREDS2) intervention study did not identify any risk of lung cancer after lutein supplementation [204,205]. Based on the evidence, it is suggested that chronic lutein supplementation at the dosage of 10 mg/day is safe and non-toxic [204,205].

## 10. Conclusions

A high intake of fruits and leafy green vegetables is important to achieve adequate dietary levels of carotenoids among other nutrients. Based on the evidence, an adequate diet is recommended rather than supplementation in order to maintain physical health. The previous finding suggests that high dietary consumption of zeaxanthin and lutein are likely to protect against ARD such as AMD. Although the beneficial effects of carotenoids for reducing the risk of ARD have been demonstrated in both in vivo and in vitro studies, there are still some controversies surrounding certain effects of carotenoids in ARD that need to be elucidated by long-term clinical trials with large cohorts of the general population. Moreover, further studies are warranted to evaluate the precise mechanism of action under pathological and healthy conditions to enhance the implementation and acceptance of carotenoids for use in clinical practice. Therefore, researchers should further investigate the underlying mechanism of action to better elucidate the possible role of carotenoids on human health.

## Figures and Tables

**Table 1:** Carotenoids content in some common foods.

Food Source	Carotenoids (µg/100 g)							
	Lutein	Zeaxanthin	Lutein and Zeaxanthin	Lycopene	α-Carotene	β-Carotene	β-Cryptoxanthin	References
Apples (with skin)	100–840				30			[40,43,44]
Apricot, raw	0–141			0.5	0–37	140–6939	28–231	[45]
Asparagus, raw	610–750				12	493		[40,44,46]
Avocados			270		28	53	36	[40,47]
Basil, raw			7050					[48]
Blackberry	270				9	100		[49]
Blueberry	230					49		[49]
Broccoli, raw	830–4300				1	414–2760		[45]
Brussels sprouts, boiled			1541					[45,47]
Carrot, raw	110–2097				530–35,833	1161–64,350		
Corn, cooked	202	202						[50]
Cress, raw	7540							[51]
Frozen corn, boiled from frozen			684					[47]
Cucumbers (with skin)	160					138		[40,44,46]
Egg whole, cooked	237	216	353					[47,50]
Egg yolk, cooked	645	587						[50]
Egg whole, raw	288	279	504					[47,50]
Egg yolk, raw	787	762	1094					[47,50]
Frozen green beans, cooked			564					[47]
Jackfruit				37–111		40–772		[52]
Kale, cooked			18,246					[47]
Leek, raw			3680					[48]
Lettuce, raw	1000–4780							[48]
Mango	100					300–4200	0–1640	[40,45]
Melon, cantaloupe					27	1595	0	[40]
Orange juice	67				8	13	34	[40,53]
Orange	64–350		129		0–400	0–500	14–1395	[40,45,47]
Orange pepper, raw		1665						[50]
Papaya	20–820			2080–4750	0–60	71–1210	60–1483	[40,45]
Parsley, raw	4326		5562					[47,50]
Peas, green, boiled			2593					[47]
Pepper, bell, green, raw	340–660				22	198	1	[54]
Pineapple						171–476		[40]
Pistachio nuts, raw			1404					[47]
Pumpkin, cooked			1014					[47]
Spinach, raw	2047–20,300		12,197			840–24,070		[40,47]
Sweet potatoes, white flesh (cooked)						25–157		[55]
Squash, boiled			2249					[47]
Strawberry	6–21					5		[40,43]
Tomato, raw	40–1300			21–62,273		36–2232		[45]
Watermelon	0–40			2300–7200	0–1	44–324	62–457	[45]
